# Implementing an mHealth system for substance use disorders in primary care: a mixed methods study of clinicians’ initial expectations and first year experiences

**DOI:** 10.1186/s12911-016-0365-5

**Published:** 2016-09-29

**Authors:** Marie-Louise Mares, David H. Gustafson, Joseph E. Glass, Andrew Quanbeck, Helene McDowell, Fiona McTavish, Amy K. Atwood, Lisa A. Marsch, Chantelle Thomas, Dhavan Shah, Randall Brown, Andrew Isham, Mary Jane Nealon, Victoria Ward

**Affiliations:** 1 Department of Communication Arts, University of Wisconsin-Madison, Madison, WI 53706 USA; 2 Center for Health Enhancement System Studies, University of Wisconsin-Madison, Madison, WI 53706 USA; 3 Group Health Research Institute, Group Health Cooperative, Seattle, WA 98101 USA; 4 Dartmouth College Geisel School of Medicine, Hanover, NH 03755 USA; 5 The Manor, Slinger, WI 53086 USA; 6 School of Journalism & Mass Communication, University of Wisconsin-Madison, Madison, WI 53706 USA; 7 Department of Family Medicine and Community Health, University of Wisconsin-Madison, Madison, WI 53715 USA; 8 Partnership Health Center, 401 W. Railroad Street, Missoula, MT 59802 USA; 9 Hunter College, New York, NY 10065 USA

**Keywords:** Addiction, Behavioral health care, mHealth, Primary care

## Abstract

**Background:**

Millions of Americans need but don’t receive treatment for substance use, and evidence suggests that addiction-focused interventions on smart phones could support their recovery. There is little research on implementation of addiction-related interventions in primary care, particularly in Federally Qualified Health Centers (FQHCs) that provide primary care to underserved populations. We used mixed methods to examine three FQHCs’ implementation of Seva, a smart-phone app that offers patients online support/discussion, health-tracking, and tools for coping with cravings, and offers clinicians information about patients’ health tracking and relapses. We examined (a) clinicians’ initial perspectives about implementing Seva, and (b) the first year of implementation at Site 1.

**Methods:**

Prior to staggered implementation at three FQHCs (Midwest city in WI vs. rural town in MT vs. metropolitan NY), interviews, meetings, and focus groups were conducted with 53 clinicians to identify core themes of initial expectations about implementation. One year into implementation at Site 1, clinicians there were re-interviewed. Their reports were supplemented by quantitative data on clinician and patient use of Seva.

**Results:**

Clinicians anticipated that Seva could help patients and make behavioral health appointments more efficient, but they were skeptical that physicians would engage with Seva (given high caseloads), and they were uncertain whether patients would use Seva. They were concerned about legal obligations for monitoring patients’ interactions online, including possible “cries for help” or inappropriate interactions. One year later at Site 1, behavioral health care providers, rather than physicians, had incorporated Seva into patient care, primarily by discussing it during appointments. Given workflow/load concerns, only a few key clinicians monitored health tracking/relapses and prompted outreach when needed; two researchers monitored the discussion board and alerted the clinic as needed. Clinician turnover/leave complicated this approach. Contrary to clinicians’ initial concerns, patients showed sustained, mutually supportive use of Seva, with few instances of misuse.

**Conclusions:**

Results suggest the value of (a) focusing implementation on behavioral health care providers rather than physicians, (b) assigning a few individuals (not necessarily clinicians) to monitor health tracking, relapses, and the discussion board, (c) anticipating turnover/leave and having designated replacements. Patients showed sustained, positive use of Seva.

**Trial registration:**

ClinicalTrials.gov (NCT01963234).

## Background

In 2013, an estimated 20 million Americans needed treatment for an alcohol or drug problem but only 11% of them received it [1]. This gap reflects both a serious shortage of funding, specialist treatment providers and treatment facilities [2, 3] and a variety of other barriers, including stigma, logistics, and denial of a substance use problem [4]. For many patients, primary care may be one of few options for help.

Federal policy encourages primary care clinics to provide behavioral health care, including management of substance use disorders [5, 6]. Federally Qualified Health Centers (FQHCs) are a key part of that initiative. FQHCs are primary care facilities (typically community health centers), required to provide care to underserved populations, offer a sliding fee scale, treat all patients regardless of ability to pay, and provide comprehensive services. Increasingly, those services include medical and behavioral/mental health care for substance use disorders [7].

Surveys of physicians indicate concerns about lack of time to deal with the complex issues surrounding addiction [8, 9] and lack of training and comfort discussing substance use with patients [10, 11]. Although behavioral health clinicians (e.g., psychologists and clinical social workers) may have more comfort and experience dealing with these issues, guidelines for behavioral health consultations in primary care suggest that most patients should be seen four times or less per year [12], which (according to traditional treatment models) is generally insufficient for those struggling with substance abuse [6].

In this context, a key question is whether mobile technology could be used in primary health care to help patients with substance use disorders. For patients, smart phones can offer continuous access to support for recovery and tools for positive behavior change, supplementing and filling in the gaps between medical and behavioral health appointments. For health-care providers, patients’ relapse/health tracking reported on the phone could inform treatment.

### The Seva Project

Seva (from the Sanskrit for “selfless caring”) is an mHealth system designed to support care for patients with substance use disorders. We received funding from the National Institute for Drug Abuse to provide Seva to three Federally Qualified Health Centers and examine effective implementation strategies (see protocol paper, [13]).

**Fig. 1:**
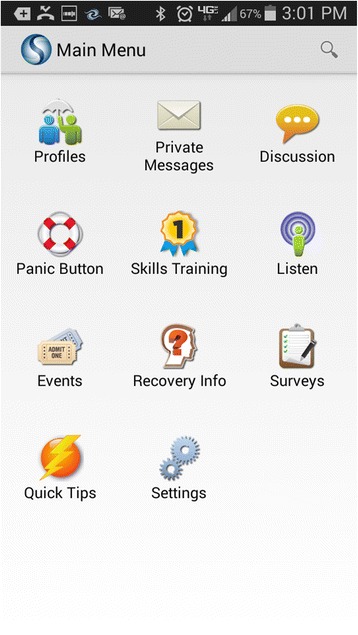
Main menu of Seva on patients’ smartphones

For the patient, Seva is an application that lives on a smart phone, with tools to support substance use treatment (see Fig. 1). The tools for patients come from two previously tested health applications: A-CHESS (Addiction-Comprehensive Health Enhancement Support System [14]) and TES (Therapeutic Education System [15]). A-CHESS resources on the phone include health tracking, a discussion board populated by patients in the study, and tools for coping with cravings and high-risk situations (e.g., relaxation exercises and links to local Twelve Step meetings). In a randomized trial in residential addiction treatment centers, patients assigned to A-CHESS had significantly fewer risky drinking days and significantly higher rates of abstinence post-discharge than those in the control group [16].

TES, the second piece incorporated into patients’ Seva app on the phone, is a web- and mobile-based curriculum for addiction treatment, comprised of 65 interactive modules designed to teach problem solving, self-regulation, coping, and lifestyle restructuring skills. When used in a model that partially replaced clinician-delivered therapy, TES produced significantly better addiction treatment outcomes, relative to a model where behavioral therapy was delivered entirely by clinicians [15, 17]. The 21 TES modules included in SEVA covered communication skills (10), triggers (3), HIV/Hepatitis (4), decision making and problem solving (4).

**Fig. 2:**
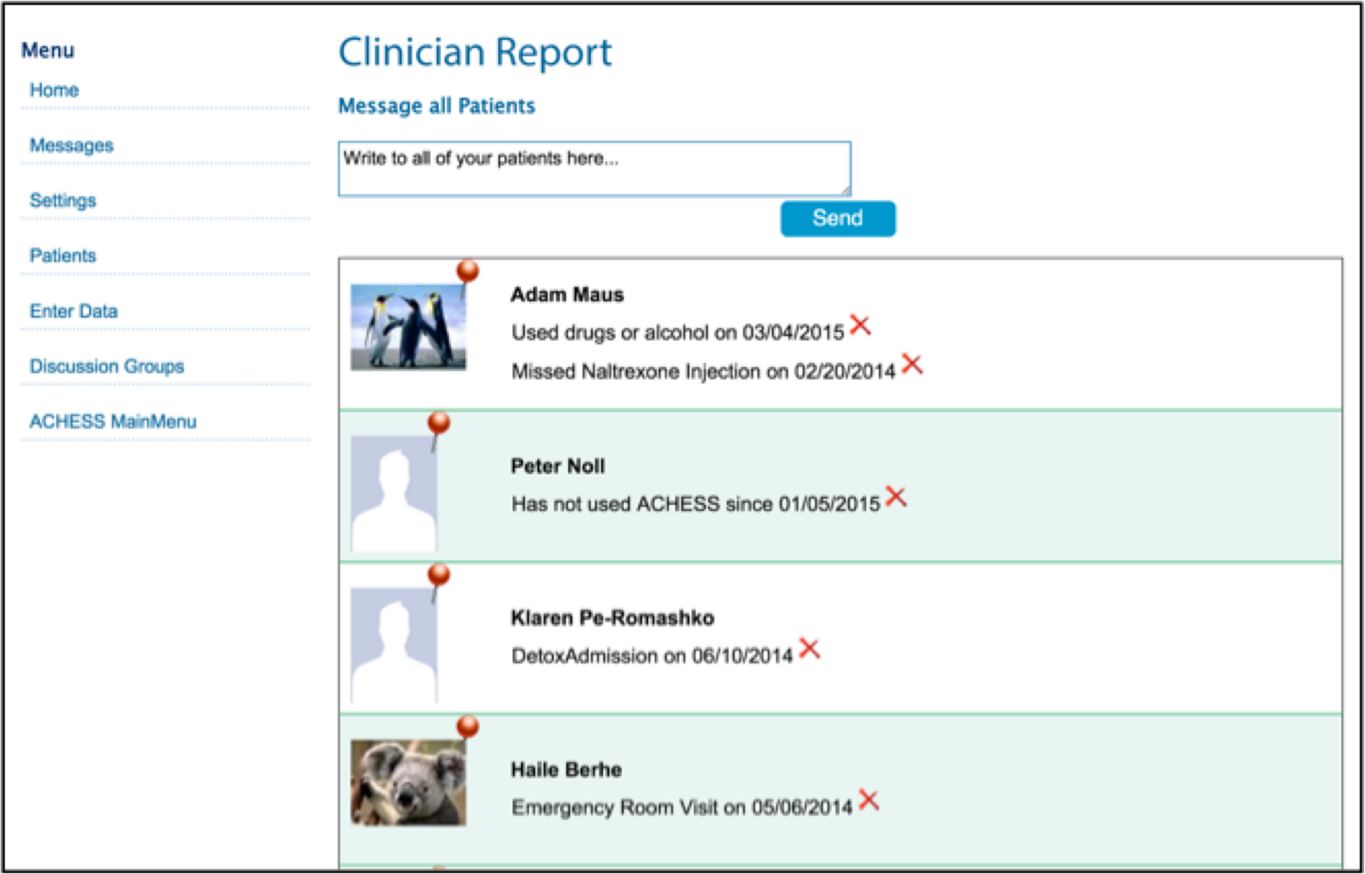
Mockup of clinician report showing hypothetical patient profiles

For clinicians, Seva provides a web portal called the Clinician Report (see Fig. 2) containing longitudinal information generated by patients’ use of Seva (e.g., weekly health-tracking surveys, including reports of relapses). Clinicians visiting the portal see a graph of participating patients’ scores over time. In a randomized trial of a cancer-related CHESS program, patients whose CHESS system included Clinician Reports (relative to those without the Report) showed significantly faster improvements in self-reported health status [18].

As shown in Table 1, we selected three very different FQHCs: the first in Madison, WI (midsize, midwest city), the second in Missoula, MT (town, rural setting), and the third in the Bronx, NY (metropolitan area). Implementation at each site was staggered at 6 months intervals. At each site, Seva is provided to 100 patients (aged 18 or older, diagnosed with substance use disorder, attended a medical and a behavioral health appointment at the site in the past year).

**Table 1:** Characteristics of research sites

	Site 1	Site 2	Site 3
	Madison, WI	Missoula, MT	Bronx, NY
	*N*	*N*	*N*
Patient Characteristics			
Number of Patients Served Prior Year	25,062	9,087	6,677
% White	60.5	91.0	3.4
% African American	25.4	1.0	32.2
% Asian	5.4	1.0	0.8
% American Indian/Alaska Native	8.2	5.0	0.4
% Hispanic	26.7	3.5	58.2
% Other		2.0	
Provider Characteristics			
*N* Medical Providers at site (MD, Resident, PA)	30	42	13
*n* Enrolled in Seva Project	3 (10 %)	25	0
*N* Nurses at site (RN, LPN, MA)	40	32	14
*n* Enrolled in Seva Project	4 (10 %)	1	1
*N* Behavioral Health at site (LMSW, LMHC, LCSW, PsyD., PhD., MD)	10	8	11
*n* Enrolled in Seva Project	10 (100 %)	4	5
Clinic Characteristics			
Medical and behavioral health care co-located	Yes	Yes	Yes
Warm handoffs to behavioral health staff	Yes	Yes	Yes
Substance use disorder focus	Medical & BHC team focused on substance use	Addiction & mental health support group	Addiction & mental health support group
Setting	Midwest US, mid-size city	Western State, small city hub for rural-frontier counties	East Coast, US Metropolitan

The first part of this paper focuses on clinicians’ initial expectations about Seva. We report qualitative data on themes that emerged at all three sites, to identify core issues that are likely to arise whenever such systems are implemented into FQHCs. The second focuses on the first year of implementation experiences at Site 1 only (given staggered implementation). We use mixed methods to describe a) implementation decisions to address clinicians’ concerns, (b) clinicians’ use and perceptions of Seva by the end of that first year, and (c) the extent to which their patients’ use of Seva was consistent with clinicians’ initial expectations.

## Methods

### Participants

#### Clinicians

As Table 1 shows, a total of 53 clinicians from three sites gave written consent to participate (i.e., to be interviewed or participate in focus groups and to have access to the Clinician Report). Two of the clinicians at Site 1 were also members of the research team. Data about clinicians’ initial expectations came from all three sites; data about experiences during the first year came from clinicians at Site 1 only (*n* = 17) given staggered implementation.

#### Patients at Site 1

A total of 120 patients were referred for participation at Site 1. Of those, 95 successfully completed the intake session (consent form and baseline survey) and subsequent training session. Of those 95 participants, 18 were dropped (e.g., lost to contact, lost multiple phones), leaving 77 at the end of the first year (*M* age = 41.6; 53.6 % female). Of these 77 participants, 38 % had a high school education or less, 46.4 % had some college or 2-year college, and 14 % had a BA or higher. With regard to race and ethnicity, 70 % identified as White, 31 % as African American, 4.3 % as American Indian or Alaskan Native, and 1 % as Hispanic. At baseline, 61.4 % of the 77 said they were very or quite comfortable using the Internet, 20.8 % said they were somewhat comfortable, and 17.7 % said they were not at all or a little bit comfortable.

### Sources of Data

#### About clinicians’ initial expectations

Data about initial expectations were gathered in the 6 months prior to implementation and in the first 2 months of implementation. As shown in the upper section of Table 2, data came from interviews and focus group with clinicians at all three sites, and notes from research team meetings that included the two clinician team members.

**Table 2:** Data collection matrix

Method/data source	Study population	Participants and number of sessions
Studying Clinicians’ Initial Expectations at All Three Sites		
Meetings/Focus Group Discussions	Clinic Staff	- Behavioral health care providers with high volume of patients with substance use disorders o Madison: 2 sessions o Missoula: 2 sessions
	Clinic Staff	- Medical &/or behavioral health care providers with varied volume of patients with substance use disorders o Madison: 2 sessions o Missoula: 1 session o Bronx: 2 sessions
In-Depth Individual Interviews	Clinic Staff	- Administration: 1–2 interviews per site - Behavioral health care providers: 1 interview per site - Medical Assistant and Nurses:1–3 per site
Research Team Meeting Notes	Clinician champions of Seva on research team	- 15 meetings of 5–9 researchers, including two clinicians from Site 1, and occasional call-ins from clinicians from Sites 2 and 3.
Studying First Year Implementation Experiences at Site 1		
Meetings toward the end of the first year	Clinic staff at first site	- Medical & behavioral health care providers with varied use of Seva: 1 session - Behavioral health care providers with varied use of Seva: 1 session
In-Depth individual interviews toward the end of the first year	Clinic staff at first site	- Physician with no use of Seva: 1 interview - Nurse with limited use of Seva: 1 interview - Behavioral health care providers with varied use of Seva: 4 interviews
Computer Data on Clinician Use of Report	Clinic staff at first site	- Log-in data from 17 clinicians participating in study
Research Team Meeting Notes	Clinician champions of Seva on research team	- 20 meetings of 5–9 researchers, including two clinicians from 1st site
Computer Data on Patient Use of Seva	Patients at first site	- Log-in data from 97 patients

*Note*. All consented clinicians or administrative stakeholders participated in at least one of the above forms of data collection. The number of participants at each session varied

#### About first year of implementation at Site 1

As the lower portion of Table 2 shows, data about *ongoing implementation decisions* came from weekly research team meetings throughout the first year that included two clinician team members from Site 1. Data about *Site 1 clinicians’ use and perceptions of Seva* 1 year into implementation came from in-depth interviews and meetings with clinicians, and quantitative data about clinician use of the Clinician report. Data about *Site 1 patients’ engagement with Seva* came from quantitative data captured every time they logged into Seva, and qualitative examination of their interactions on the Seva discussion board. As patients were informed during the consent process, all of their activity on Seva is captured and stored in a database. For privacy reasons, no data are gathered about non-Seva uses of the phone, such as text messaging or phone calls.

#### Data collection

In all but two instances, meetings, interviews, and focus groups were not audio-recorded. Rather, the lead author took detailed notes as a form of quasi-transcription. After each session, she went through the notes to make additions and corrections and to organize the material. She then summarized key findings or themes and disseminated them to the research team to critique and verify the synthesis. To verify the accuracy and completeness of this approach, two sessions were audio-recorded. Comparison of coding from the detailed notes versus transcripts from audio recordings of these two sessions indicated no differences.

#### Data analysis

Notes and transcripts were analyzed using thematic analysis. The lead author identified key sections to be coded, developed a coding scheme, and coded all key sections. A second coder (a student) independently coded a random selection of 4 research team meetings and 4 in-depth interviews. Krippendorff’s alpha was above .80 for all themes.

## Results

### Clinicians’ Initial Expectations: Data from All Three Sites

The aim was to identify core themes regarding clinicians’ expectations about Seva that were evident at all three research sites-issues that were raised consistently, despite differences in the clinics' geographic setting, organizational structure, etc. Some were predictable concerns about workflow and workload that often arise at implementations in healthcare settings [e.g., [19]]. Other concerns and anticipated benefits are unique to the issue of using an mHealth intervention in primary care to support management of substance use disorders. Table 3 summarizes core concerns in order of number of clinicians who independently raised each theme. Table 4 summarizes anticipated benefits. The numbers under-estimate support for each theme, given that they do not reflect clinicians who nodded agreement during meetings or focus groups.

**Table 3:** Core themes in clinicians’ initial concerns about implementing Seva

Qualitative Themes (*N* Independent Mentions)	Exemplar Quotations
Concerns about workflow and time	
Fitting it in to the workflow (10)	We’re so busy here. What’s the right workflow? How do we interface the systems? Don’t make me log in to another system, don’t send me an email attachment, don’t make me open a document. How will this fit into the huddle?
Difficult to engage physicians (8)	I’ve talked to [some doctors] but they’re pretty overwhelmed right now… Medical providers have so many pieces they use right now, I just don’t think that they would log on.
Having time for Seva (7)	I’m in a storm and can’t really see out of the storm. I worry about having another thing added if I don’t get extra time carved out for it.
Encourage needy patients (4)	I worry that this is going to increase burden on staff. Some of these patients are in bad shape and out of control. I worry they will use the phones to hound the staff even more.
Other initiatives compete for time & energy (3)	A lot of things start to happen and then don’t stick. Some pan out and some don’t… We were going to be involved in a brain mapping system and that didn’t pan out because of funding and logistics. And we’re very close to contracting with a casino upstate to be gambling treatment providers. And, 2015, we have a big depression care initiative. And we have to meet the demands of all these licensing bodies.
Concerns about legal obligations & liability	
Possible unanswered suicidality on the discussion board (5)	I could be held liable. I could lose my license. I am uncomfortable with the idea of giving out the phone and not getting this information directly…People who are not me making clinical decisions about my patients. If one of our patients were to do something self-injurious, I would be thoroughly investigated, and this is never far from my mind.
Patients understand what clinicians can see (4)	Particularly when there are possible disclosures about substance use that have not previously been shared with the medical team and place them at risk based on their current medication regimen (someone is disclosing heavy benzo use while on suboxone for one example)… I want to be upfront for their protection and for ours-it’s my license if I’m documenting stuff.
Concerns about patients’ use of Seva	
Not use Seva or misuse the phone (6)	My biggest fear is patients not using or misusing the system. That they’d just be signing up to get a free phone and then they’d be pawning it or that they’d throw it in the river.
Toxic interactions on discussion board (6)	Particularly when individuals are reaching out during low moments, their pre-existing negative emotional valence may be inadvertently infused into their interpretation of the messages and statements they are reading, particularly if the messages are ambiguous, have multiple meanings, or are written poorly.

**Table 4:** Core themes in clinicians’ initial expectations of benefits of implementing Seva

Qualitative Themes (*N* independent mentions)	Exemplar Quotations
Seva as a resource for patients	
With few other sobriety resources (6)	Missoula has nothing long term for patients with substance use issues. There’s only one 4-bed share house. Turning Point, the only outpatient program has a long waiting list. There’s nothing else in town. People basically have AA or [the clinic’s] sobriety group. And for people who live further out of town in a small community, or on the reservations? This is something we can offer them.
As a tool for learning and insight (5)	Their lack of language to talk about emotions is really profound. They can’t explain what happened, don’t know how to tune into different feelings, so they turn to substances. A powerful part of the process is teaching them how to recognize emotions and providing them with options. So just filling out the BAM [Brief Alcohol Monitoring Scale… on Seva] is a powerful tool.
Who need an alternative to group meetings (5)	Often they want to be alone-they’re often living in shelters that are very chaotic, and they just want peace and quiet…. So Seva would allow them to interact without really being part of a group. They can get their toe in the water.
To experience constant availability of sobriety support (4)	Often a patient is trying to reach out to me but I’m busy and won’t get the message till five hours later. I really like that in the meantime, the phone can help them with their breathing exercises or he can listen to a podcast to help him figure out why he shouldn’t relapse, and that’s great.
At key transitions (4)	This has so many positive possibilities. Like being able to help people coming out of rehab, or from mental health inpatient, or coming out of jail and they need that support to help them now they’re back in the community.
Clinician Report as a resource for clinicians	
More efficient appointments (10)	If they can be filling out the PHQ [Patient Health Questionnaire] ahead of time on the phone, and if I can see that, that saves me a ton of time. That can make our meetings a lot more efficient.
Prompt primary care conversations about addiction (6)	Addictions are so often kept secretive in a medical visit, being able to talk about it is really important. If we could give it to the medical provider, it would be really good. It could start the conversation.
Mobile phone as a resource	
For patients (5)	Having a phone helps them move into housing, they can call the hotline phone number at the homeless shelter, people can be calling in for help with issues.
For clinic (5)	So many of these patients don’t have voicemail, don’t have a phone system. Now suddenly we can access them. It suddenly lets us have contact… so we could remind them, “oh you’ve got this appointment” or we can reach out, “hey, just checking in.”

### Initial Concerns

#### Concerns about clinician time and workflow

As Table 3 shows, the most frequently mentioned concern was about workflow (when/how to check the Clinician Report, issues of interface with the Electronic Medical Record and logging in) and having adequate time assigned to Seva monitoring and patient outreach. Clinic administrators and behavioral health clinicians were particularly concerned that physicians would not engage with Seva, given high case-loads. At Sites 1 and 2 (midWest city, rural city respectively), physicians thought the Report looked interesting, but were dubious they would use it for the small subset of their patients with substance use disorders, particularly if it required a separate log in. At Site 3, the clinic did not introduce Seva to physicians due to these concerns.

#### Concerns about whether and how patients would use Seva

Clinicians noted the complex mental and physical co-morbidities often facing this patient population, and were concerned whether patients would learn how to use Seva, and whether some would sell the smartphone, lose it, or break it. Additionally, they wondered whether patients would use the discussion board inappropriately (e.g., engage in hostile interactions or try to sell or buy drugs).

#### Concerns about legal obligations and liability

Clinicians wanted to be certain that patients would understand that their healthcare providers could see anything they did on Seva. This was particularly an issue given that patients’ disclosures on Seva about relapses could affect their treatment (e.g., clinicians’ decisions about medications). Additionally, clinicians were concerned that patients might use Seva to indicate that they were feeling suicidal (or wanted to hurt someone) and that the clinic (or they themselves) would be liable if no-one responded. No site wanted to be responsible for monitoring patients’ posts or health-tracking responses.

### Expectations of Benefits

#### Seva as a resource for patients

As shown in Table 4, the most commonly anticipated benefit of Seva was as a resource for high need patients facing key transitions (e.g., coming out of jail), or who had few other sources of sobriety support (e.g., “had burned their bridges”), or who needed alternatives to group meetings such as AA/NA meetings or group therapy. Some individuals had mental health issues that made it difficult for them to benefit from group interactions; some lived in isolated communities which made it embarrassing and stressful to attend local sobriety support groups. The constant accessibility of Seva on the phone was seen as a way for patients to experience continuity of sobriety support between appointments, or at moments when clinicians were not immediately able to respond.

#### Clinician Report as a resource

Behavioral health clinicians anticipated that their appointments and outreach could become more efficient, given access to patients’ health tracking scores. Additionally, clinic administrators and behavioral health care providers noted that if physicians did use Seva, it might increase their comfort discussing addiction with patients and increase their referrals to behavioral health.

#### The mobile phone as a resource

The fact that Seva is offered on a mobile phone (as opposed to other platforms) was seen as beneficial in various ways. Clinicians noted that some patients with substance use disorders have volatile living situations, including frequent changes in address and loss of phone service. Providing them with a stable phone number could facilitate appointment reminders and case management between appointments, and might reduce patient attrition. It could also help patients find critical resources (e.g., homeless shelters) or reach out to crisis hotlines. Additionally, offering the smartphone to patients who are often highly stigmatized could serve as a sign of trust and respect.

### First Year of Implementation Experiences: Data from Site 1

#### The clinic context

Several features of Site 1 are particularly relevant to the implementation of an addiction-targeted mHealth system. First, behavioral health care is integrated with medical care-they are situated in the same building, and if physicians learn that a patient has substance use issues they can have the patient meet a behavioral health care provider immediately (“a warm handoff”). Second, the clinic has a multi-disciplinary “Health Promotions” team with expertise in health care needs of patients at risk for adverse consequences of tobacco, alcohol, prescribed controlled substances, and other drugs. Given this, responsibility for addressing issues related to risky and problem substance use and psychoactive medications often falls on behavioral health and on Health Promotions. Third, two clinicians from Site 1 are members of the Seva research team and serve as champions of the project at the clinic. They identified the initial set of patients for recruitment, facilitated intake sessions, were the first to use the Clinician Report for patient care, disseminated information from the Clinician Report to other clinicians, and provided near-weekly feedback about implementation issues as they arose.

### Implementation Decisions During the First Year at Site 1

Various strategies evolved over the first year, in response to clinicians’ initial concerns.

#### In response to concerns about monitoring

Clinicians wanted assurances that someone would monitor the discussion board daily, but given their workload did not want to do so themselves. It was agreed that two non-clinic members of the research team would monitor the board daily, remove any inappropriate content, contact the patient and their behavioral health care provider as needed, and write responses to patients so that no-one was left unanswered.

#### In response to concerns about liability

Given clinician concerns about transparency, patients were explicitly told that their healthcare team could see all their responses on Seva. Participants specifically consented to exchanges of information about their substance use between the researchers and healthcare providers. Behavioral health clinicians often discussed Seva during appointments, further highlighting the potential visibility of their responses.

Given clinician concerns about liability if patients expressed suicidal or dangerous ideation, the intake and training sessions emphasized that Seva was not a way to reach clinic staff in an emergency. Seva itself included on-screen text: “If you are in crisis or thinking about harming yourself, please call (number for suicide hotline)”. This message showed on-screen whenever the patient hit the panic button, or responded negatively to the daily query about making it through the day. The two research team members who monitored the discussion board contacted relevant behavioral health clinicians if patients indicated they were in crisis.

#### In response to concerns about logging in to Clinician Report

Clinicians were initially concerned about the inconvenience of logging in to the Clinician Report, and particularly skeptical that physicians would take the time to do so. Over the first year, the clinic developed a model in which a few clinicians agreed to log in once or twice a week and share actionable information with other behavioral health clinicians so that outreach could take place if needed.

### Clinician Use of the Clinician Report

At the end of the first year of implementation (with 11 months of Clinician Report availability), 5 of the 17 clinicians had never accessed the Clinician Report, 9 showed very limited use (*M* = 3.56 days total logged in during 11 months), and 3 showed extensive use (*M* = 71.68 days logged in during 11 months). The most extensive use was by a behavioral health care provider assigned to check the Report and share actionable items (e.g., relapses) with other behavioral health care providers. The two clinicians who were members of the research team also logged in frequently and disseminated information to other clinicians. Additionally, toward the end of the first year, two clinicians (one nurse, and the behavioral health research team member) set up email alerts so they would be notified directly if a patient on Seva reported a relapse (i.e., reported using drugs or alcohol).

Although this evolving system avoided the burden associated with everyone logging in to the Report, it was susceptible to personnel change. The behavioral health consultant assigned to check the Report weekly left the clinic, as did the nurse who signed up for email alerts. One of the two champions of Seva took maternity leave for part of the year. This placed more emphasis on the research team members who monitored the discussion board to act as information hubs.

### Clinician Perceptions of Seva

Toward the end of the first year, clinicians were asked about their perceptions of Seva. Their responses were coded with regard to the core themes from the pre-implementation phase.

#### As a resource for patient care

Clinicians on the addiction-focused Health Promotions Team, and behavioral health care providers perceived that many of the anticipated benefits of Seva (reported 1 year earlier) had been realized. Although most of them did not log in to the Clinician Report, they reported discussing Seva with patients during appointments, including encouraging use of specific features (e.g., the learning modules and relaxation podcasts). All said that their patients valued the constant accessibility of support. As anticipated, Seva provided an alternative for patients who (for various reasons) did not want to attend group therapy and/or were not interested in various other forms of treatment. A behavioral health care provider noted,

“Several people who are using it the most are people who’ve failed out of other treatments or burned out on people telling them how to change. They really appreciate the autonomy to choose as much or as little as is really helpful.”

Clinicians also noted that patients were learning from the educational material on Seva.

“One thing that’s really interesting is to look at language they’re picking up from the modules. I had one man who didn’t have much vocabulary for insight before, and I recently heard him talking about self-sabotage and triggers, and he got that from the modules.”

The main exception to benefits anticipated and achieved for patients was the possibility that patient-generated data on Seva would help primary care physicians discuss substance use with their patients. As one physician reported,

“ To be honest, some of my patients are using Seva and I’ve never even seen them. I know they’re on it because I get a notification, as their PCP… but they’re typically pretty healthy and haven’t had other PCP needs… or I’m not usually talking about it with them because I’m talking about other stuff.”

Thus, Seva was part of treatment for substance use disorders at the clinic, but it was implemented by the addiction-focused Health Promotions team and behavioral health care providers, rather than primary care physicians.

#### Perceived impact on workload and flow

When asked about the impact of Seva on their work load and flow, the behavioral health care providers noted the substantial burden added by the tasks of recruiting patients and coordinating intake and training sessions (“but I was okay with that, because I was committed to the project”). Among the few who regularly logged in to the Clinician Report, there was clearly some effort involved, but also a sense of routine.

It’s not really making more work. I try to log into Seva when I start my shift, unless I get really slammed at the start of the shift. I try to keep up with the Discussion Board. I look to see who has used [alcohol or drugs] recently and whether they’ve had other support from us. If they have used and we haven’t seen them in a while, I will give them a call.

Among those who did not log in to the Clinician Report (i.e., received Seva information from colleagues designated to log in), the response was very positive.

“Oh no, Seva makes less work, it doesn’t make more. Once we figured it out, it makes it easier to help patients, we knew we had extra thing for them. It’s really something helpful, an extra resource.”

At the same time, those behavioral health consultants who did not access the Clinician Report saw little likelihood that they would do so in the future. As anticipated, they felt it was too hard to fit in to their established routines and busy schedule.

“I just haven’t figured out a way to incorporate it into the workflow. I rely on [specified clinicians] reaching out to me, like they’ll tell me that there’s a post that’s concerning around safety and risk. I just haven’t used it, just because of the workflow issues. I think when you’re trying to change workflow habits, that’s really hard.”

Despite their initial concerns, they did not perceive that patients became more demanding and needy as a result of having access to Seva and a study-provided smartphone.

#### Minimal concerns about liability

Although some clinicians had initially expressed concerns about liability, at follow-up they did not have such concerns. They felt confident that the discussion board was being monitored and that patients understood what could be seen.

### Evidence about Patients’ Use of Seva

Clinicians were initially somewhat dubious about patients’ use of Seva and the phone. One year later, data about patients’ patterns of use speaks to these issues.

#### Lost and broken phones

An initial concern was that patients would lose, sell, or break their phones. Of 97 patients enrolled, 14 patients lost or broke their phone. Of these, half were replaced because the patients had shown high levels of engagement with Seva.

#### Levels of engagement

**Fig. 3:**
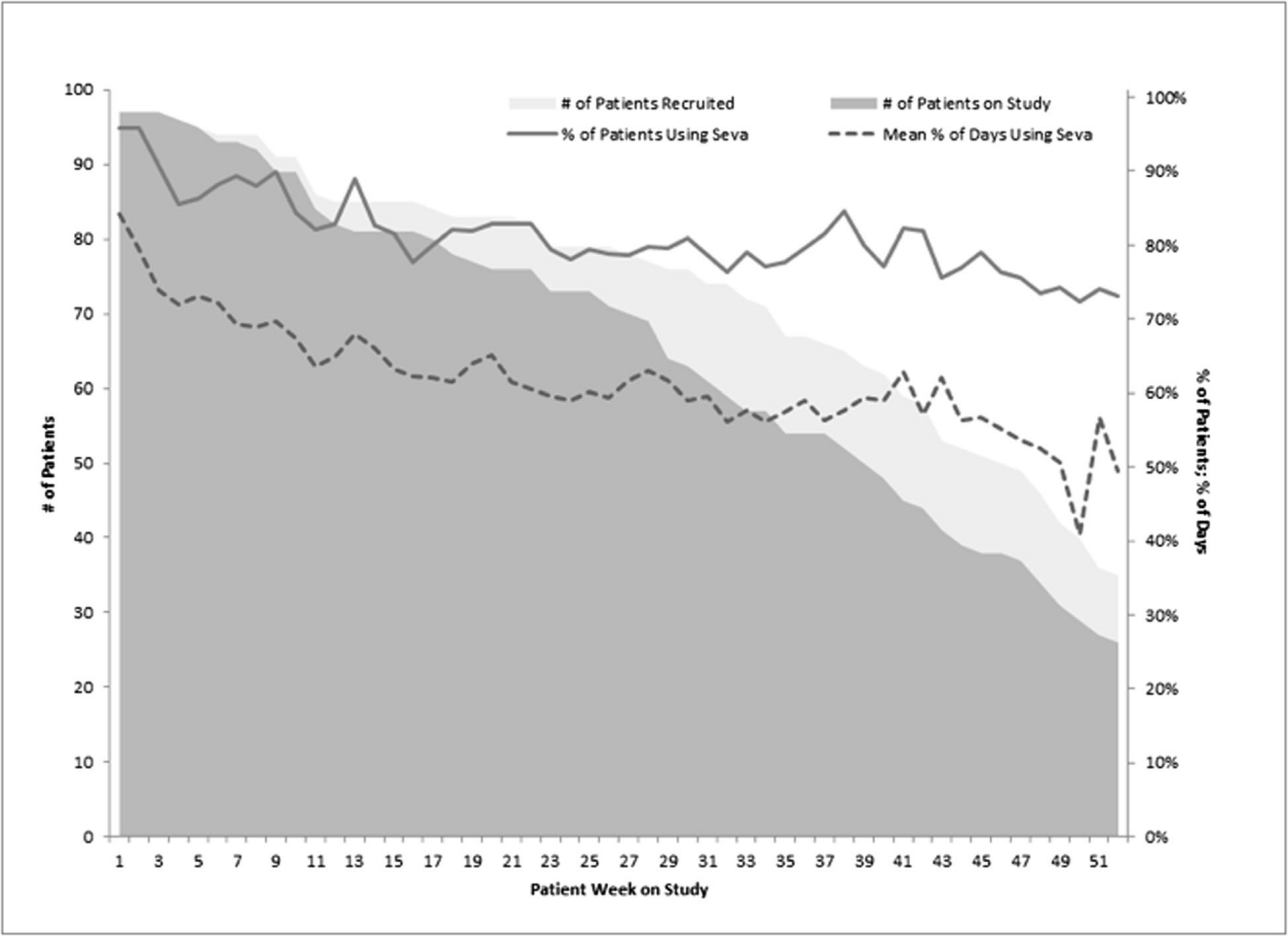
Patients’ use of Seva at site 1, reported by patients’ week on study

Clinicians wondered whether their patients would actually engage with Seva, or “just sign up to get a free phone.” As can be seen in Fig. 3, 80 % or more of participants at Site 1 logged on at least once per week for the first 20 weeks that they were on the study (not including time spent on training). During weeks 21–41, the percent logging in continued to be high (70–92 %) and the median days per week logging in remained above 5.5.

#### Interactions on the discussion board

Clinicians initially expressed concern that patients might use the discussion board inappropriately. Hundreds of messages were posted over the course of the year, and there were few problematic incidents. One involved an increasingly hostile series of posts between two participants over whether it was necessary to report a relapse. A third participant posted a warning that they were risking access to Seva and sent a private message to the research team, asking them to step in. In a second instance, a participant complained about a frustrating day, and asked (in an ambiguously joking way) if anyone knew where she could buy heroin. Other participants promptly posted rebukes (“not cool”) and contacted the researchers. In both cases, the problematic content was removed, the individuals were warned privately by the support team, and continued on study without further incident. Thus, the group engaged in constructive and effective self-policing, combined with daily monitoring and responses from the two appointed members of the research team.

## Discussion

At all three sites, clinicians’ initial concerns focused on workload and workflow, but also on issues of whether patients with substance use disorders would engage with Seva and use it appropriately, and issues of clinic liability if patients’ use of Seva were not adequately monitored.

During the first year of implementation at Site 1, a number of strategies emerged to mitigate these concerns. Seva was used by behavioral health care providers and the addiction-focused Health Promotions team, not by physicians. A few key clinicians undertook the task of checking the Clinician Report and sharing information with others as needed. This strategy reduced burden, but was problematic when those key individuals took leave or took jobs elsewhere. Concerns about liability for unanswered “cries for help” on Seva and possible inappropriate use of Seva were addressed by having two members of the research team (rather than clinicians) monitor the discussion board and contact the clinic as needed.

Initially, clinicians at all three sites perceived that patients with substance use disorders were a high-need population that would benefit from the continuous access to social support and recovery tools on Seva. After a year of implementation, behavioral health care providers at Site 1 felt that their patients were indeed benefiting. Although there were some losses of the phone, patients showed high levels of sustained engagement with Seva and appropriate, supportive use of the discussion board.

### Limitations

It remains to be seen whether these experiences and strategies change over time and whether they are shared by the other two clinics. These are primarily qualitative data, reflecting clinicians’ self-reported experiences and perceptions.

## Conclusions

MHealth interventions for substance use disorders may face some issues during implementation in primary care settings. These include clinic concerns about who will monitor patients’ interactions online, clinicians’ lack of time to monitor patients’ online health tracking, and the reluctance of physicians to engage with such a system. Nonetheless, the current study also indicates that behavioral health clinicians perceived substantial benefits of such an intervention for their patients and that the patients showed sustained use of it over the year, with supportive, appropriate interactions.

## Abbreviations

FQHC: Federally Qualified Health Center
